# Exploring the Effects of Climate Variability on Diarrheal Diseases, Malaria, and Malnutrition in Children Under Five Years in Revuè Sub-Basin, Manica Province, Mozambique

**DOI:** 10.3390/tropicalmed11090258

**Published:** 2026-09-12

**Authors:** Tatiana J. Marrufo, Genito A. Maúre, Américo F. José, Osvaldo F. Inlamea, Bettencourt P. S. Capece, Maria-Raquel G. Silva

**Affiliations:** 1 Faculty of Science and Technology, Fernando Pessoa University, 4249-004 Porto, Portugal; 2 Instituto Nacional de Saúde, Marracuene 020502, Maputo, Mozambique; americo.jose@ins.gov.mz (A.F.J.); osvaldo.inlamea@ins.gov.mz (O.F.I.); 3 RISE-Health, Faculty of Health Sciences, Fernando Pessoa University, Fernando Pessoa Teaching and Culture Foundation, 4249-004 Porto, Portugal; raquel@ufp.edu.pt; 4 Department of Physics, Faculty of Science, Eduardo Mondlane University Main Campus, Av. Julius Nyerere 3453, Maputo 0101-11, Mozambique; genito.maure@uem.ac.mz; 5 Faculty of Veterinary, Eduardo Mondlane University, Av. de Moçambique, Km 1,5, Maputo 0104-11, Mozambique; bpscapece@yahoo.com; 6 FP-I3ID, FP-BHS, Faculty of Health Sciences, University Fernando Pessoa, 4200-150 Porto, Portugal; 7 Molecular Oncology and Viral Pathology Group, Research Center of IPO-Porto (CI-IPOP) &RISE@CI-IPOP (Health Research Network), Portuguese Oncology Institute of Porto (IPO-Porto)/Porto Comprehensive Cancer Center Raquel Seruca (Porto. CCC), 4200-072 Porto, Portugal; 8 CHRC, Comprehensive Health Research Centre—Group of Sleep, Chronobiology and Sleep Disorders, Nova Medical School, Nova University of Lisbon, 1150-090 Lisbon, Portugal; 9 CIAS, Research Centre for Anthropology and Health—Group of Human Biology, Health and Society, University of Coimbra, 3000-456 Coimbra, Portugal

**Keywords:** climate variability, diarrheal diseases, malaria, malnutrition, ecological study, Manica

## Abstract

Transmission of water, vector, and foodborne diseases tends to increase with climate variability, disproportionately affecting vulnerable communities. Globally, this dynamic contributes to adverse health outcomes in low- and middle-income countries such as Mozambique. Nevertheless, the influence of climate variability on disease patterns remains unclear. We assessed the potential relationship between climate variables and diseases, including diarrheal diseases, malaria, and malnutrition, in four districts around the Revuè sub-basin. An ecological, longitudinal, retrospective study conducted from 2016 to 2024 used secondary weekly disease data and annual malnutrition records. We applied a Generalized Additive Model framework to evaluate non-linear associations between climate factors and disease case counts. Our main findings showed that while higher maximum temperature was associated with increased cumulative malaria case counts, cumulative and heavy rainfall (~100 mm/week) was associated with decreased diarrheal disease case counts. Higher prevalence of moderate acute malnutrition is associated with a decrease in annual mean total precipitation (*p* = 0.0062). In contrast, higher prevalence of severe acute malnutrition is associated with a decrease in annual mean temperature (*p* = 0.0312). This study contributes to understanding climate variables associated with water, vector, and foodborne diseases, enhancing our understanding of factors contributing to overall food insecurity and affecting public health.

## 1. Introduction

The Intergovernmental Panel on Climate Change (IPCC) defines [1] climate change as the change in the state of the climate that can be identified by changes in the mean and/or the variability of its properties and that persists for an extended period, typically decades or longer. Climate variability, meanwhile, is defined by IPCC 2022 as variations in the mean state and other statistics of the climate on all spatial and temporal scales beyond that of individual weather events [1]. Both events are intensifying worldwide, exerting profound effects on human health and well-being. They contribute to the global burden of disease and extend their impact on food security [2]. These dynamics disproportionately affect vulnerable populations, particularly through the transmission of water-borne, vector-borne, and foodborne diseases. Such outcomes emerge from disruptions affecting interconnected systems including water, environment, food, and human ecosystems [2,3,4].

Anthropogenic emissions of greenhouse gases, including carbon dioxide, methane and nitrous oxide, contribute to long-term climate change, including rising temperatures, changes in precipitation patterns and extreme events, all of which pose significant threats to public health [3,5]. The World Health Organization (WHO), ) estimates that by 2050, the consequences of climate change and climate-sensitive diseases may result in an additional 250,000 deaths per year. In the context of high-emission scenarios, the projected mortality burden is anticipated to escalate to more than 9 million additional deaths per year by 2100 [6].

The global water crisis currently affects more than 700 million people, who lack access to clean water [,^7^]. This crisis is reflected in both the quality and quantity of surface water resources, as well as in the increasing demand for groundwater [8] noting the relationship between climate change and water resource degradation primarily driven by hydrological events that significantly reduce water quality and availability, thereby impacting both natural and human systems. Floods represent a climate-related hydrological event that disrupts water supplies, heightens morbidity and mortality from infectious diseases and malnutrition, and exacerbates food insecurity in low- and middle-income countries (LMICs) [9,10]. Climate change has been demonstrated to exert an effect on water ecosystems through alterations in air temperature, precipitation, and wind patterns. These changes have been shown to have a subsequent impact on terrestrial ecosystems and to impose limitations on the expansion of human populations [7,11]. It is a well-documented fact that in Africa, post-flooding outbreaks of diarrheal diseases including cholera, typhoid, and dysentery have been frequently reported with microbial agents including *Cryptosporidium* spp., *Giardia duodenalis*, and *Salmonella* spp. [12]. In addition to malaria, the burden of acute diarrheal diseases, particularly cholera, is expected to worsen under projected climate scenarios [13]. The duration of floods and droughts, humidity levels, temperature fluctuations, and seasonal shifts in rainfall distribution are key factors associated with the prevalence of waterborne diseases [13,14,15,16]. The vulnerability of populations to these diseases is further compounded by a few factors, including poverty, low literacy rates, limited access to healthcare, widespread malnutrition, dietary challenges, and exposure to both water and air pollution [12].

Climatic impacts and ecological degradation are major drivers of biodiversity loss, with significant implications for interactions between wildlife and human populations. These processes have the potential to modify the habitats of disease vectors, facilitating the spread of zoonotic and vector-borne diseases into new regions and settings [13,15,17]. Malaria, the most significant vector-borne disease associated with climate hazards, is endemic across much of Africa. Variations in temperature, precipitation, biodiversity loss, and shifts in seasonal duration generate optimal conditions for the proliferation of mosquitoes [4,13,17,18]. Recent studies have indicated that other vector-borne diseases, including dengue and chikungunya, may be exacerbated by demographic shifts associated with climate variability, particularly within urban environments [15]. These dynamics have been shown to increase the costs of prevention [19] and management [13] of vector-borne diseases, thereby further straining public health systems in the context of ongoing environmental and climate crises.

In Sub-Saharan Africa (SSA) and South Asia, climate change is intensifying the risk of food insecurity and malnutrition. Extreme weather events have a significant impact on availability, with the destruction of crops resulting in an increase in demand and subsequent disruption to food systems [16]. In these regions, prolonged reductions in food intake led to chronic malnutrition and stunting among children, rendering them more vulnerable to infectious diseases and increasing mortality within this group [20]. Prolonged droughts, rising temperatures, and delays in the onset of the monsoon season are among the primary drivers of childhood malnutrition, with direct consequences for children’s mortality [18,21].

Cumulative environmental hazards, including but not limited to water scarcity, biodiversity loss, crop yield, artisanal mining pollutants, and climate variability, are compounding vulnerabilities in Mozambique. The Revuè sub-basin is of particular concern in this regard, as it is experiencing a heightened burden of climate-sensitive infectious diseases, including malaria and diarrheal diseases, as well as malnutrition [22,23]. This has resulted in an increased demand for health systems, necessitating the development of effective strategies to address these challenges. It is important to characterize how climate variability is associated with health outcomes in this setting. For this reason, this study describes the patterns of infectious diseases and malnutrition in Manica’s districts from 2016 to 2024; the dynamics of malnutrition using survey-based indicators measured up to 2023; and analyzes infectious diseases and malnutrition outcomes in relation to climate variability along the sub-basin of Revuè in Manica.

## 2. Materials and Methods

### 2.1. Study Area and Design

The Revuè River sub-basin is in Manica Province, central Mozambique, encompassing an area of approximately 4400 square kilometers. The river originates in the mountainous regions of the Manica District, an area characterized by the Manica Greenstone Belt, which is rich in alluvial gold deposits. The Revuè River and its tributaries flow through this geologically significant area, within the Manica District, extending into Macate, Sussundenga, and Vanduzi adjacent districts. The basin’s location and environmental conditions, including soil properties, are conducive to the growth of crops used to sustain the region’s population [24].

This study employs a retrospective ecological design to investigate the association between climate variability and infectious disease case counts, as well as outcomes related to them in Manica over nine years between 2016 and 2024. The study integrates epidemiological and climate datasets to assess the impact of temperature, rainfall and humidity on malaria and diarrheal disease trends, including the relation with malnutrition outcomes.

### 2.2. Data Variables, Sources, and Processing

Infectious disease data includes weekly reported cases of malaria and diarrheal diseases stratified by district, collected from the Weekly Epidemiological Bulletin. Acute malnutrition data includes annual reported cases of moderate and severe malnutrition stratified by district. Data obtained from District-Health Information System (DHIS-2) [25] and hosted by the Ministry of Health were focused on children under five. The data on disease surveillance were available only as raw case counts reported weekly by district through the national health information system. This raw data used in the construction of models reflects the actual reporting structure, thereby ensuring that assumptions were not introduced using smoothing or imputation. These raw counts provide a transparent basis for exploratory analysis of temporal and spatial patterns, particularly in settings where denominators are uncertain or inconsistently available. Household malnutrition indicators using standardized Z-scores of WHO Growth Standards [26], which reflected child malnutrition (stunting, underweight, and wasting), were obtained from Family Budget Surveys (FBS, 2019 and 2022) [27,], and the Demographic Health Survey 2022–2023 [28], and reported data are presented as percentages.

Climate data include hourly total precipitation (mm), air temperature (°C), and air relative humidity (%) that were provided by the European Center for Medium-Range Weather Forecasts (ECMWF) with a spatial resolution of 0.25 degrees (~25 km) and have been converted to weekly data to match the timeline of weekly malaria and diarrheal disease data. Also, annual mean climatic variables (temperature and precipitation) between 2019 and 2023 were collected from Provincial Statistics Yearbooks [29,30,31,32] to match the timeline of annual acute malnutrition data. A summary of the variable definitions and respective sources is presented in Table A1 of Appendix A.

Temporal aggregation of climate and disease (malaria and diarrhea) data was made at the weekly level for consistency. Since climatic influences on infectious diseases may exhibit delayed effects, climate variables were lagged by 0–12 weeks, with moving averages assessed and allowing for testing the seasonality effect. Disease outcomes were modeled as weekly raw case counts to reflect the actual reporting structure without the introduction of a population offset, allowing for a transparent evaluation of temporal and spatial count patterns. Malnutrition data were calculated as annual cases per 1000 population, with district population estimates from the National Institute of Statistics [33].

### 2.3. Sample Size

The total sample size of the data was determined based on the total number of observations for each type of variable analyzed during the period of 2016–2024.

### 2.4. Statistical Analysis

Generalized Additive Models (GAMs) [34] were used to assess the non-linear associations between climate factors and infectious disease case counts (malaria and diarrheal diseases). To model the over-dispersed weekly infectious disease counts, we employed a Negative Binomial error distribution with a logarithmic link function. A Distributed Lag Non-Linear Model (DLNM) [35] was integrated into the GAM framework to assess lagged effects of climate exposures across lag periods of 0–12 weeks, based on biological plausibility and the prior literature on incubation and transmission cycles [3,4,5]. The exact fitted model equation is specified as:

log(E[*Y_t_*]) = α + cb(*x_t_*, lag) + *s* (*t*, bs = ‘’cr’’, k = 16) + *District_i_*

where *Y_t_* is the weekly count of reported disease cases at time *t*; α is the model intercept; and cb(*x_t_*, lag) represents the cross-basis matrix for the climate predictor *x* and its lagged effects. The exposure–response function was parameterized using cubic regression splines (fun = “cr”), and the lag–response function was parameterized using natural cubic splines with 3 degrees of freedom (fun = “ns”, df = 3); *s*(*t*) is a smooth function of calendar time to control for long-term trends and seasonality, utilizing a cubic regression spline basis (bs = “cr”) with the basis dimension set to k = 16; and *District_i_* is a categorical fixed effect to account for baseline spatial differences across the four districts.

While a population offset was not included to model true incidence rates, baseline demographic and spatial heterogeneities between the four districts (such as varying average population sizes) were accounted for by including the district as a categorical fixed effect in the model. Relative risks (RRs) were calculated by centering the cross-basis splines at the median value of each respective climate variable. The optimal degree of smoothing and model penalty selection was performed using Restricted Maximum Likelihood (REML), which provides robust variance component estimates for negative binomial GAMs. To evaluate model fit and rule out residual autocorrelation, we inspected partial autocorrelation function (PACF) plots and deviance residuals.

For acute malnutrition outcomes, specifically moderate acute malnutrition (MAM) and severe acute malnutrition (SAM), parametric GAM and mixed-effects models were discarded due to the limited number of annual observations (2016–2023 across two districts with continuous series, Manica and Sussundenga). Instead, we relied strictly on non-parametric Spearman rank correlations (ρ) [36] as an exploratory analysis to assess monotonic relationships between annual climate variables and malnutrition prevalence. This non-parametric approach is robust to small sample sizes and outliers without risking overparameterization. Statistical significance was established at *p* < 0.05. All statistical analyses were conducted in R Statistical Software (version 4.5.0) using the mgcv package (version 1.9-1) for GAM fitting and the dlnm package (version 2.4.7) for cross-basis evaluation. Some charts were improved in Microsoft Excel.

### 2.5. Ethical Considerations

Ethical approval was sought from Mozambique’s National Health Bioethics Committee (reference 024/CNBS/24).

## 3. Results

### 3.1. Infectious Diseases and Climatic Variables

Table 1 summarizes the associations between weekly meteorological indicators and the relative risk of diarrheal diseases and malaria in children under five. Moderate temperatures are significantly associated with a higher risk of diarrheal disease. The temperature of 20 °C was found to have a higher relative risk (RR = 1.25; 95% CI: 1.05–1.45). The relative humidity was at approximately 60%, which was also associated with an elevated risk (RR = 1.20; 95% CI: 1.00–1.40). Precipitation with light rainfall (defined as 50 mm or less) exhibited an increase in risk (RR: 1.05; 95% CI: 0.90–1.20), while heavy rainfall resulted in a reduction in risk. The lagged responses were minimal, with the risk of diarrheal diseases showing slight variation beyond 5–10 weeks (e.g., Tmean lag RR: 0.99; 95% CI: 0.96–1.02). Only Tmean was statistically significant in relation to diarrheal diseases. For malaria, the relative risk at 36 °C (RR = 1.15; 95% CI: 0.90–1.45) was found to be higher. The relative humidity levels ranging between 75 and 80% were associated with a reduced risk, with RR = 0.90; 95% CI:0.75–1.05. Intense precipitation (>100 mm) was significantly associated with an elevated risk (RR = 1.25; 95% CI: 1.10–1.40). The presence of lagged effects was indicated, with an increase in malaria-reported weekly case counts observed after a period of exposure for Tmax, TP and RH, and lasting between five and seven weeks (e.g., Tmax lag RR = 1.08; 95% CI: 1.01–1.15).

**Figure A1:**
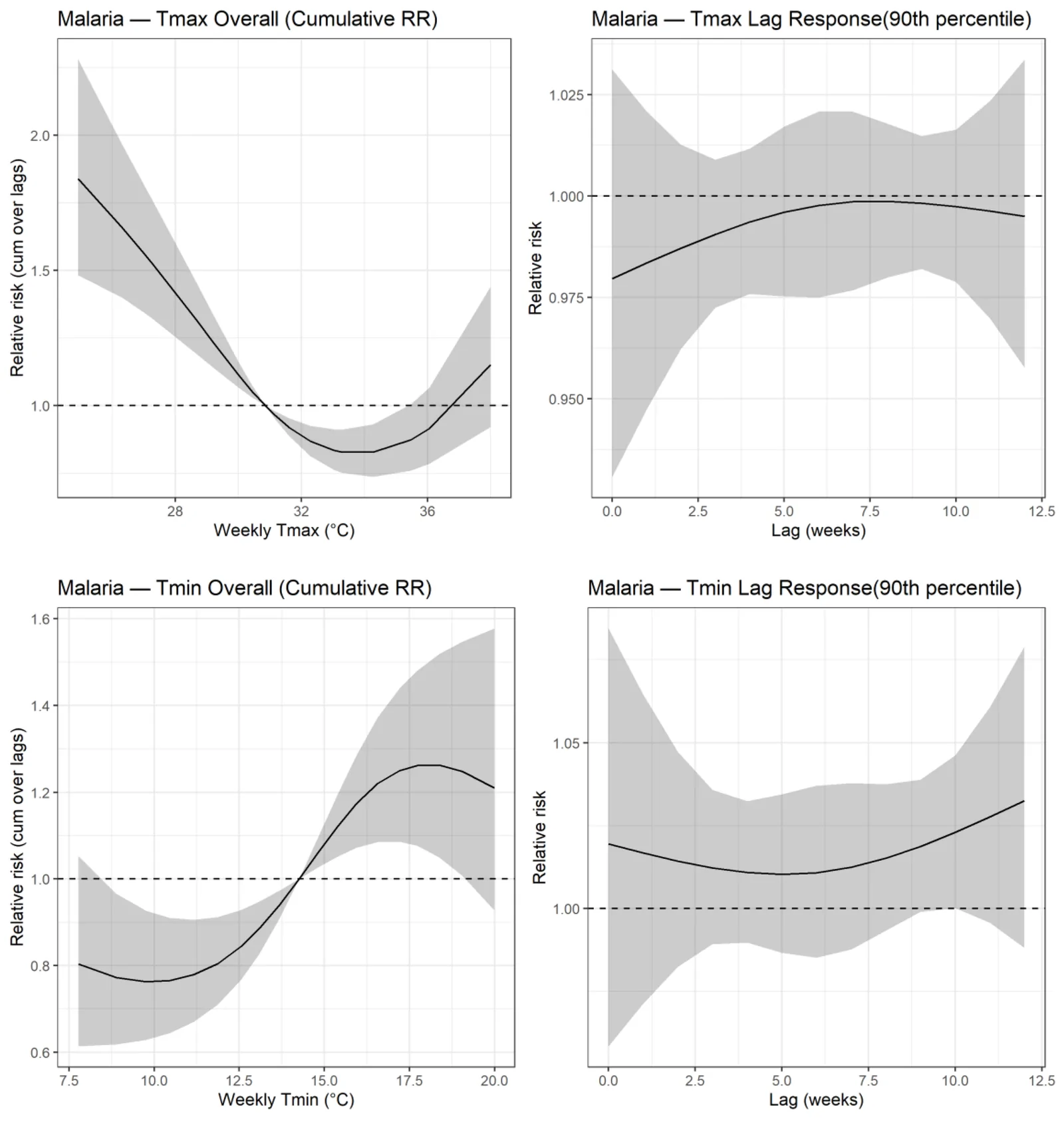

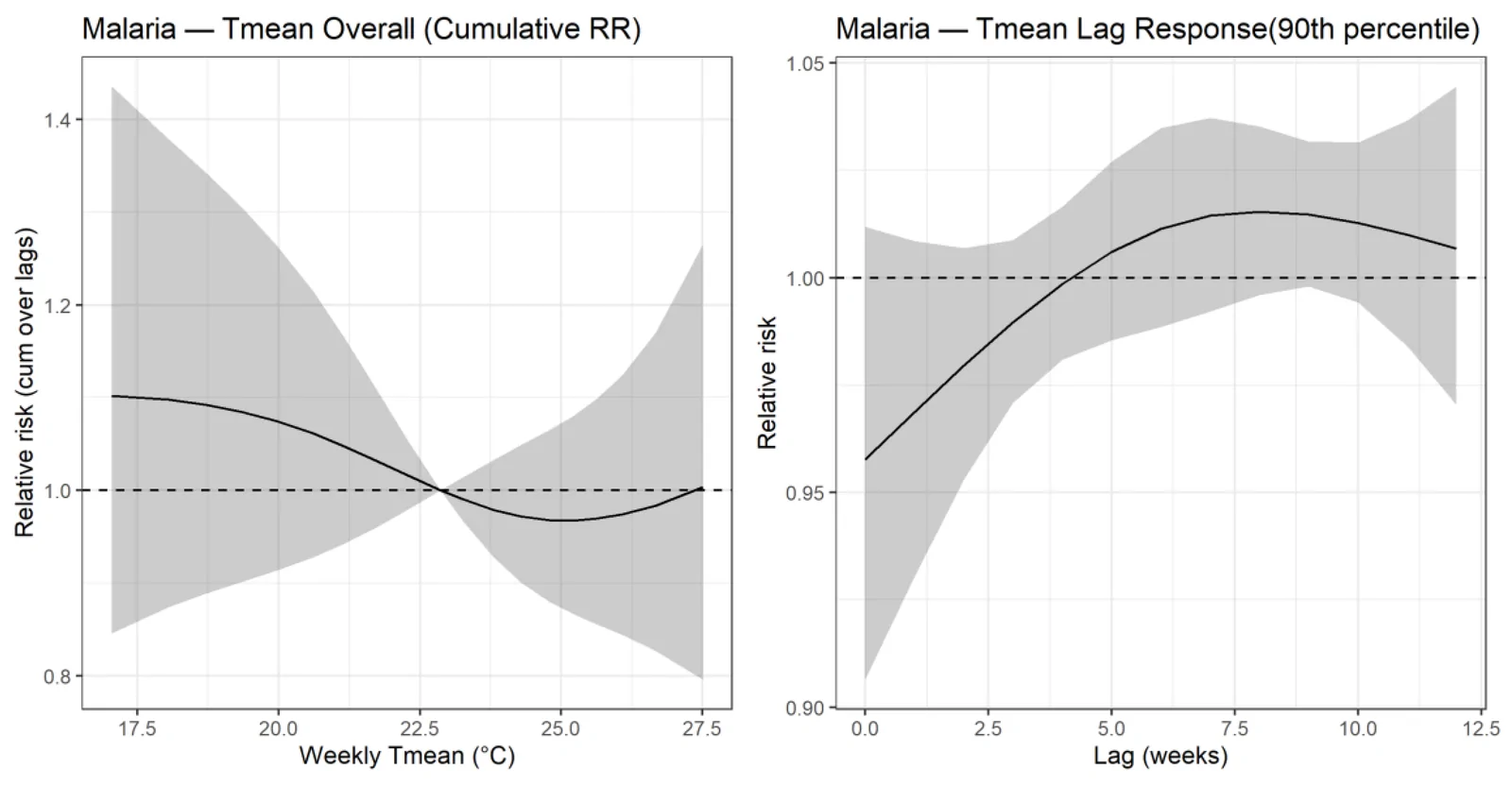
Exposure–lag–response surfaces from the DLNM showing the effects of minimum, maximum, and mean temperature on malaria case counts.

**Figure A2:**
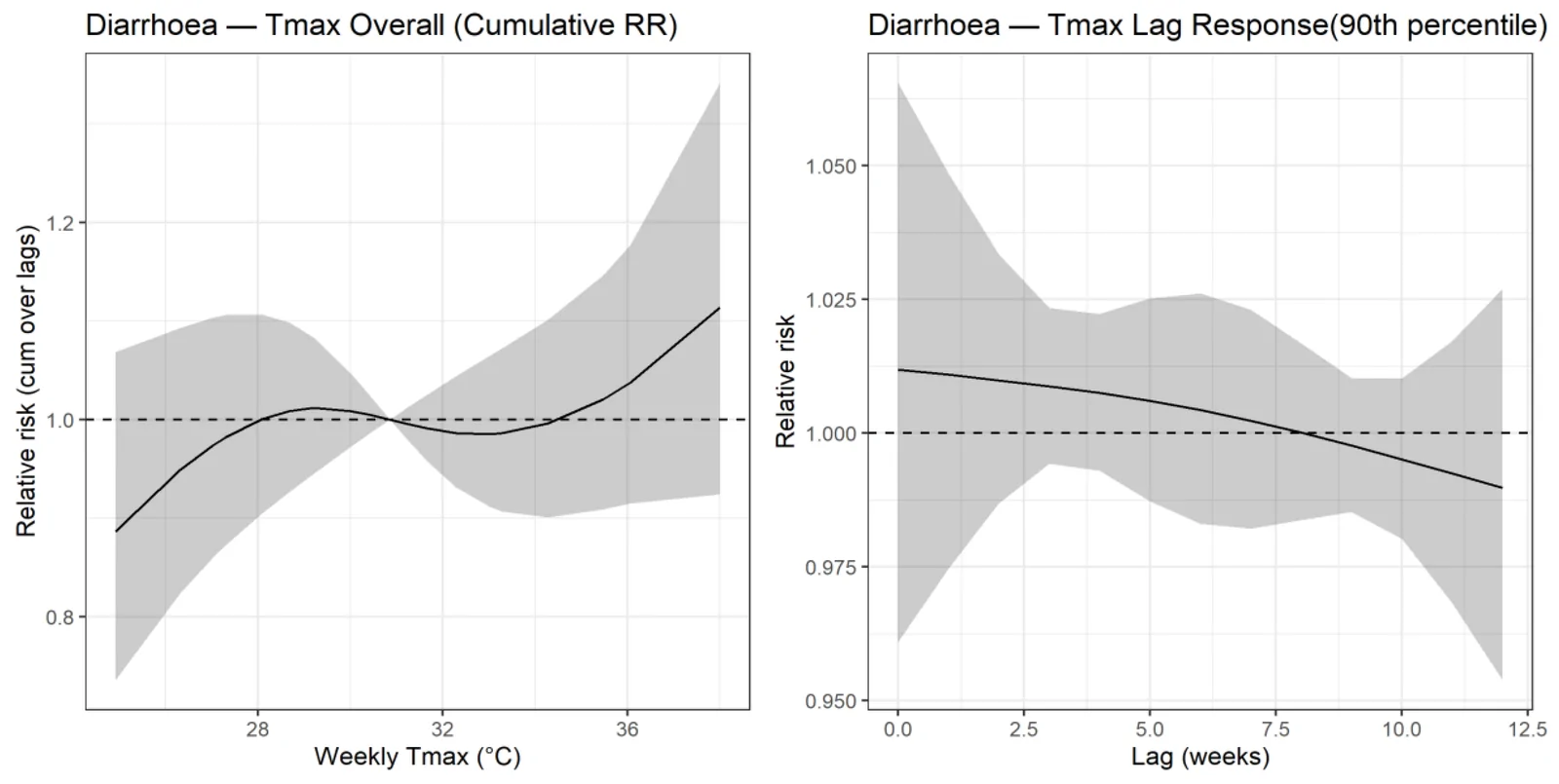

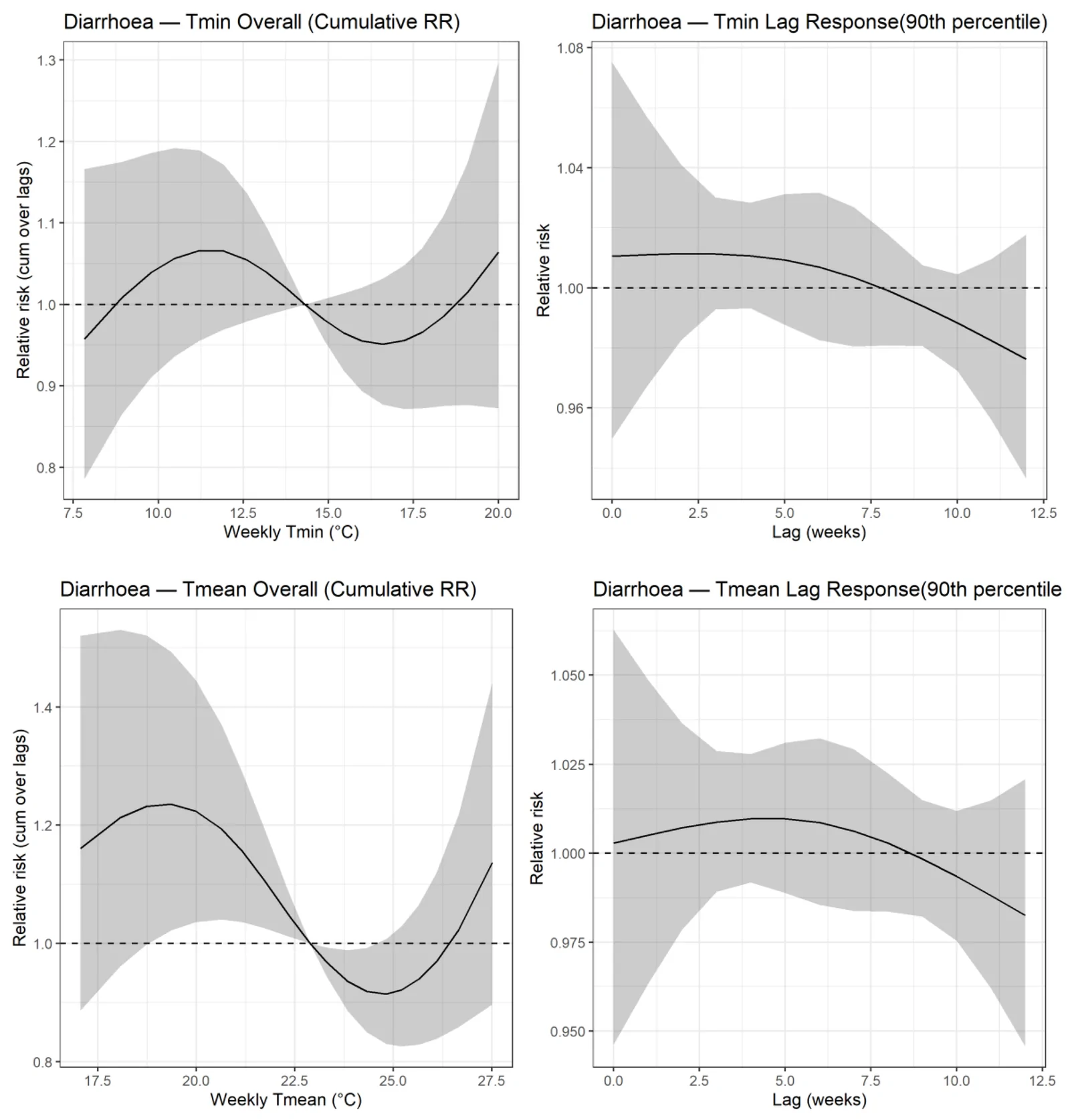
Exposure–lag–response surfaces from the DLNM showing the effects of minimum, maximum, and mean temperature on diarrheal disease case counts.

**Figure A3:**
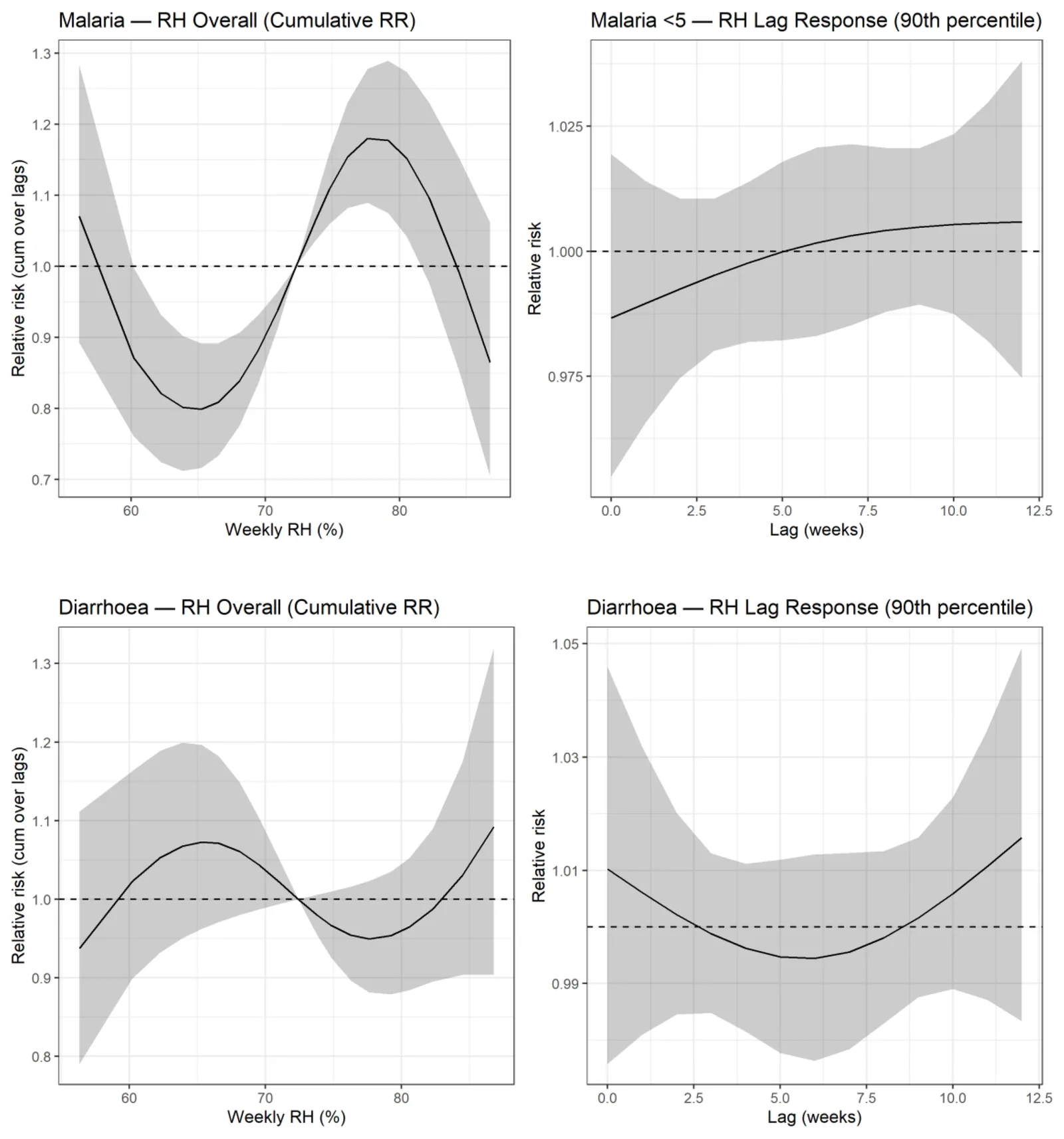
Exposure–lag–response surfaces from the DLNM showing the effects of relative humidity, on both malaria and diarrheal disease case counts.

**Figure A4:**
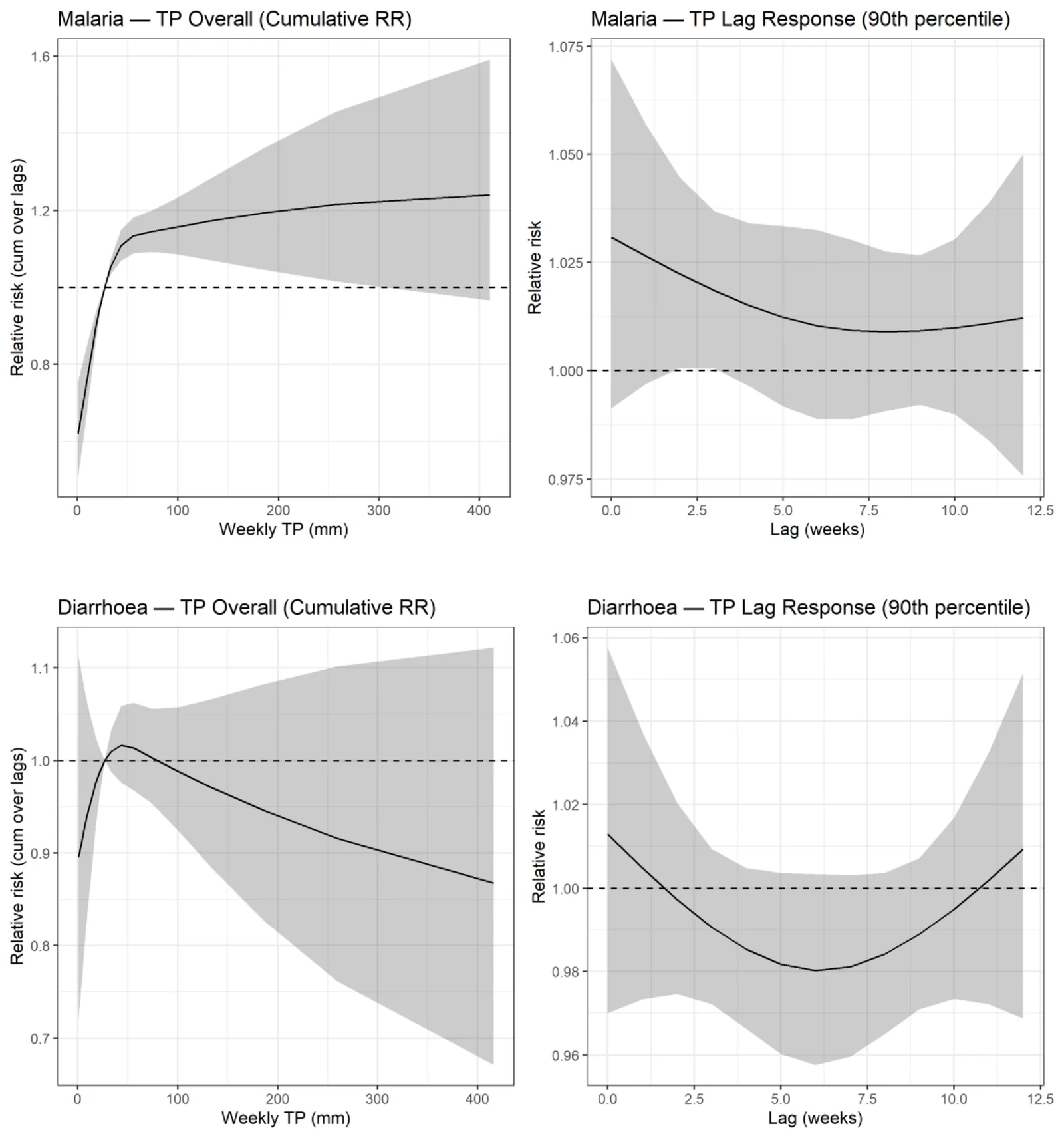
Exposure–lag–response surfaces from the DLNM showing the effects of total precipitation on both malaria and diarrheal disease case counts.

In Appendix B, Figure A1, Figure A2, Figure A3 and Figure A4, the plots herein demonstrate the relative risk of infectious diseases in relation to the observed climatic variables. The plots constitute the initial stage of the development of our DLNMs, the findings of which are summarized in Table 1 below.

### 3.2. Malnutrition Outcomes and Climatic Variables

**Figure 1:**
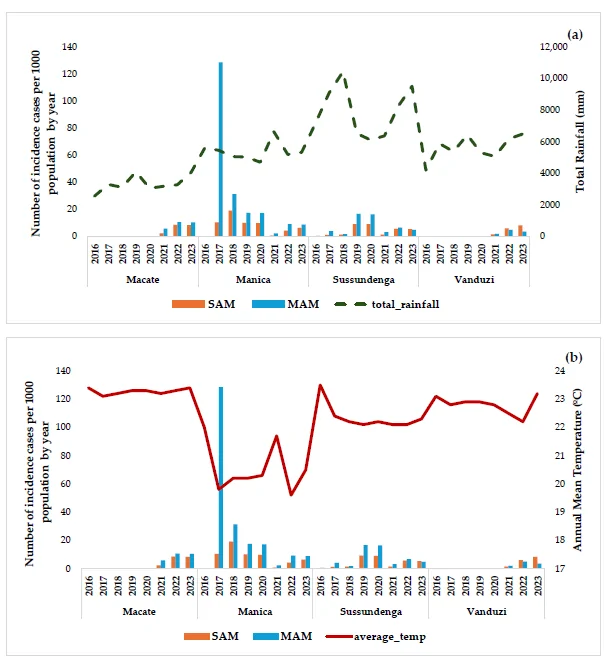
Trends of moderate and severe acute malnutrition incidence rate by year across Manica districts and behavior of total precipitation (**a**) and mean temperature (**b**) between 2016 and 2023.

Trends in acute malnutrition, both severe acute malnutrition (SAM) and moderate acute malnutrition (MAM), were analyzed in the four districts of Manica Province for the period 2016–2023, as shown in Figure 1. The districts of Manica and Sussundenga had the highest incidence rates of both MAM and SAM over the years, with MAM predominant in Manica in 2017 and 2018, and in Sussundenga in 2019 and 2020. The highest incidence was 128 cases per one thousand population at risk of MAM in Manica. Total precipitation in Sussundenga varied between 5300 and 10,400 mm across the years, whereas in the Manica district, the variation was lower, between 4700 and 6600 mm. Regarding annual mean temperature, variation was observed in Manica ranging from 19.6 to 21.7 °C, while in Sussundenga the observed variation was lower, ranging from 22.3 to 23.5 °C. The districts of Macate and Vanduzi reported sporadic cases between 2016 and 2020. Since 2021, Macate has exhibited an increasing trend in both MAM and SAM, reaching a total of approximately eleven cases per one thousand population at risk. In contrast, Vanduzi showed an increasing trend in both outcomes until 2022, followed by a divergent pattern. MAM decreased to around two cases per one thousand population at risk, while SAM increased from 6 to 8 cases per one thousand population at risk between 2022 and 2023. Precipitation in Macate increased steadily over the years, ranging from 2500 to 4000 mm. In contrast, Vanduzi experienced inconsistent precipitation levels ranging from 4200 to 6400 mm. The mean temperature in these two districts was consistently above 23 °C.

**Figure 2:**
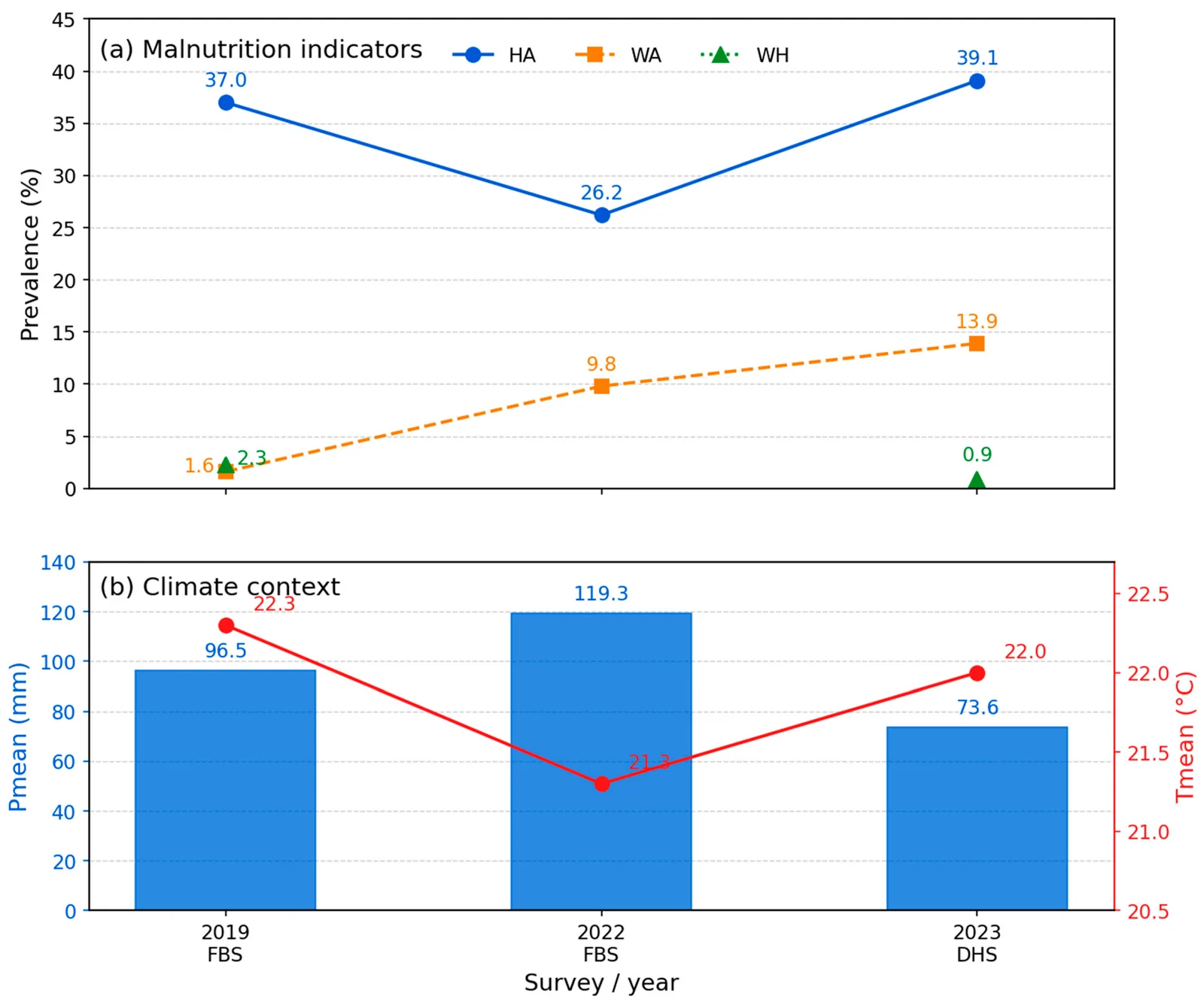
Temporal variation in malnutrition indicators from national surveys and corresponding patterns in provincial annual mean precipitation and temperature. HA, prevalence of height-for-age (stunting); WA, prevalence of weight-for-age (underweight); WH, prevalence of weight-for-height (wasting); FBS, Family Budget Survey; DHS, Demographic and Health Survey; Pmean, annual mean precipitation (mm); Tmean, annual mean temperature (°C); mm, millimeters; and °C, degrees Celsius.

According to the national surveys reported in Manica province between 2019 and 2023, stunting (HA) is the most prevalent malnutrition outcome as per Figure 2. This indicator exhibits an upward trend among children under five between 2022 and 2023, with a registered mean prevalence ranging from 26.2 ± 1.2 to 39.1 ± 1.8. The next most prevalent outcome is underweight (WA), which has shown an increasing trend over the years, reaching a mean prevalence of 13.9 ± 0.3. Wasting (WH) is the least prevalent form of malnutrition reported throughout the years, with the highest mean prevalence observed in 2019 at 2.3 ± 1.0. Figure 2 demonstrates the temporal variation in malnutrition indicators and corresponding climatic variations, where annual mean temperature decreased from 2019 to 2022, then increased in 2023 to a range of 21.3–22.3 °C. Concurrently, the annual mean total precipitation exhibited an increase from 2019 to 2022, subsequently decreasing in 2023 to a range of 73.6–119.3 mm.

### 3.3. Modeling Infectious Diseases and Climatic Variability

**Figure 3:**
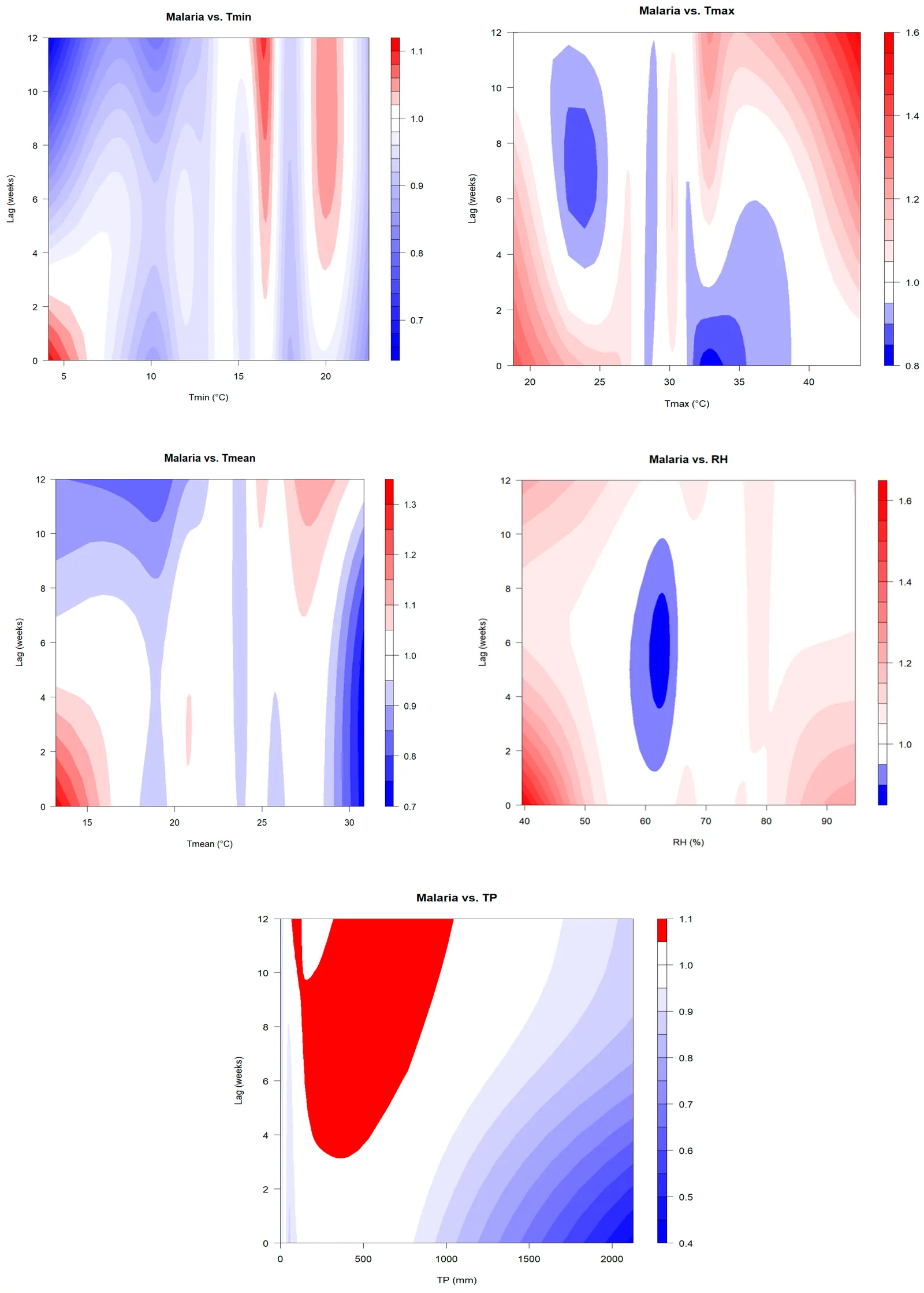
Distributed lag non-linear model surfaces illustrating the combined exposure–response and lag–response relationships between climatic variables (minimum, maximum, and mean temperature; relative humidity; and total precipitation) and malaria case counts. Abbreviations: RH—relative humidity; Tmin—minimum temperature; Tmax—maximum temperature; Tmean—mean temperature; and TP—total precipitation.

**Figure 4:**
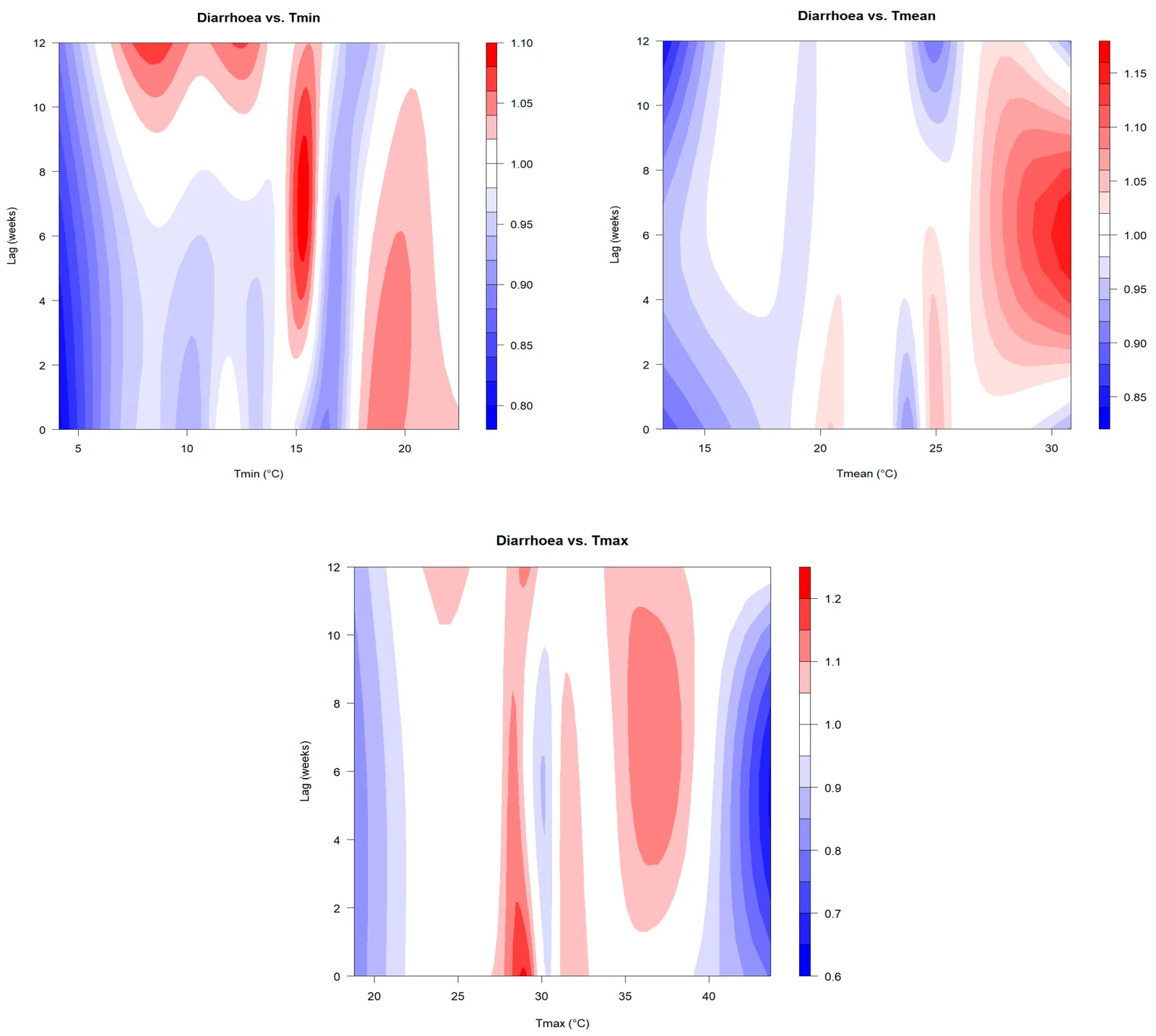

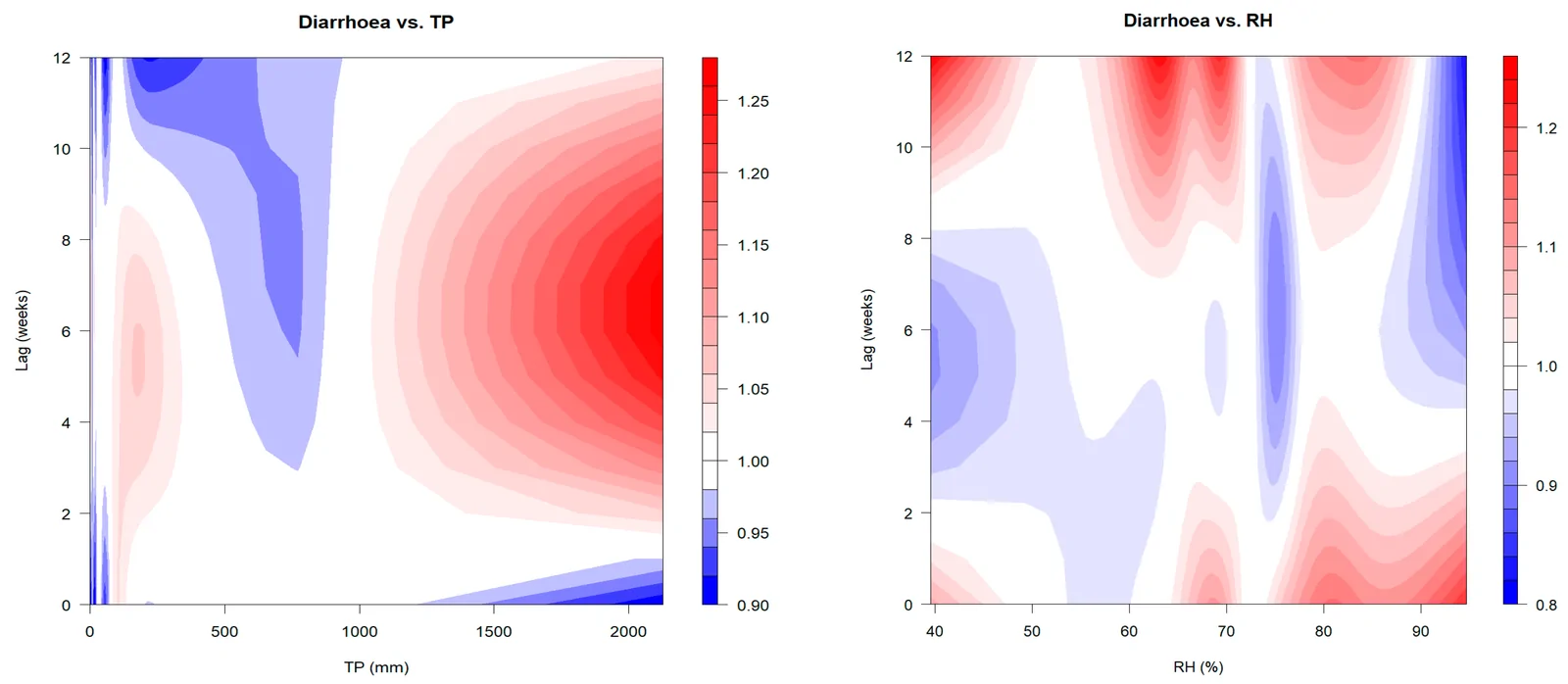
Distributed lag non-linear model surfaces illustrating the combined exposure–response and lag–response relationships between climatic variables (minimum, maximum, and mean temperature; relative humidity; and total precipitation) and diarrheal disease case counts. Abbreviations: RH—relative humidity; Tmin—minimum temperature; Tmax—maximum temperature; Tmean—mean temperature; and TP—total precipitation.

To contextualize the specific vulnerabilities of young children, the analysis produced simplified contour plots for children under five of malaria and diarrheal diseases against each climate predictor, as shown in Figure 3 and Figure 4, respectively. The contour plots indicate that minimum temperature is associated with a delayed increase in malaria-reported case counts, beginning around week four, when the RR rises above 1.0. A positive and delayed correlation is evident between maximum temperature and malaria case counts. As maximum temperature rises, the logarithm of malaria cases increases concomitantly, indicating higher risk at warmer temperatures, as illustrated in Figure 3.

This association is particularly pronounced at the upper temperature ranges, where malaria case counts intensify. A lag–response effect is evident for maximum temperature, with malaria case counts increasing from week six onwards, as RR climbs from 1.2 to 1.6. In contrast, relative humidity exhibited a divergent pattern with malaria case counts reaching 40–50% relative humidity just before week two and subsequently declining in subsequent weeks. Conversely, at 55–65% relative humidity, the negative correlation strengthened steadily from week one to week ten, and delayed significant malaria-reported weekly case counts from week zero to six when relative humidity was above 80%. The rainfall–malaria association was non-linear and lag-dependent. Table 1 showed elevated precipitation-associated risk in the summary and cumulative estimates, whereas the DLNM surface suggested attenuation at some higher-rainfall/lag combinations. These patterns should not be interpreted as a simple positive or inverse correlation.

The contour plot in Figure 4 illustrates the relationship between the case counts of diarrheal disease and the temporal dynamics of climatic factors. A heightened risk of diarrheal diseases manifests at temperatures ranging from approximately 15 °C to 20 °C, with a latency period of several weeks, indicating that a decline in minimum temperatures may precede an escalation in the cumulative case counts of the condition after a delay. At higher minimum temperatures, the risk remains lower or stable across children under five, with minimal lagged effects. The likelihood of experiencing diarrheal diseases increases in conjunction with maximum temperature, particularly within a range between 27 °C and 36 °C, exhibiting a delay of several weeks (zero to twelve). For the mean temperature, negative associations predominate at values below 18 °C, with a delay of several weeks, whereas positive and significant risk emerges at higher temperatures (≥27 °C) from week 1 through week 11. The relative humidity demonstrates a distinct pattern: as the humidity levels rise to between 65 and 95 percent, the relative risk of diarrheal diseases becomes more pronounced during weeks 8 and 12. The probability of the risk of diarrheal diseases in relation to precipitation is positive, but not significant, particularly from week three to ten, at higher volumes of rainfall.

### 3.4. Modeling Malnutrition Outcomes and Climatic Variability

**Figure 5:**
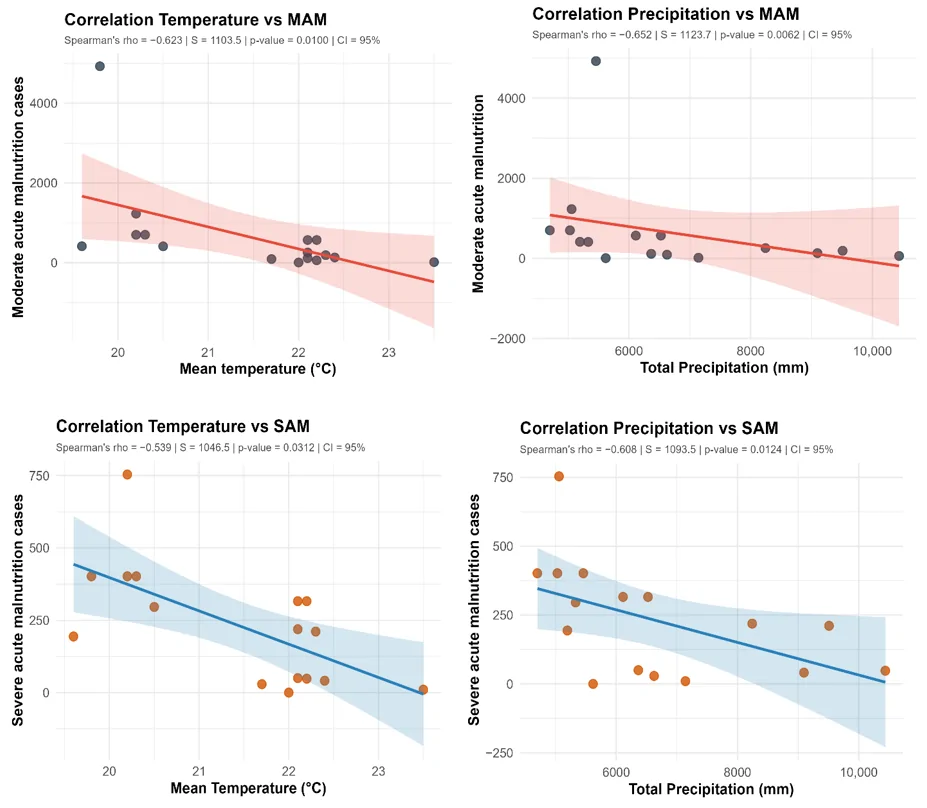
Correlation plots illustrate the association between moderate and severe acute malnutrition, and mean temperature and total precipitation among the districts of Manica and Sussundenga. Abbreviations: CI—confidence interval; MAM: moderate acute malnutrition; and SAM: severe acute malnutrition.

Because of the interrupted cases in the given period analyzed, the interannual variation in acute malnutrition was considered only for the combination of districts with continuous series of data among years, namely Manica and Sussundenga (Figure 1). The DLNM and GAM-spline models were discarded for malnutrition outcomes due to the limited number of years available. Across the two eligible districts (Manica and Sussundenga) over the 2016–2023 period, our effective sample size was *n* = 16 annual observations (df = 14), providing exploratory estimates, as illustrated in Figure 5. For moderate acute malnutrition, a significant negative correlation was identified between mean temperature (Spearman’s ρ = −0.623, *p* = 0.0100, *n* = 16, df = 14), indicating that higher annual average temperatures were associated with a reduced number of reported cases among children under five. Similarly, total precipitation demonstrated a substantial negative correlation with acute moderate malnutrition (Spearman’s ρ = −0.652, *p* = 0.0062, *n* = 16, df = 14). For severe acute malnutrition, a significant negative correlation was observed between total precipitation (Spearman’s ρ = −0.608, *p* = 0.0124, *n* = 16, df = 14), indicating that higher annual rainfall tends to report fewer cases of severe acute malnutrition among children under five. Conversely, temperature exhibited a moderate negative correlation with severe acute malnutrition (Spearman’s ρ = −0.539, *p* = 0.0312, *n* = 16, df = 14).

## 4. Discussion

This ecological study conducted in central Mozambique highlights the complex interplay between climate variability and health outcomes in local communities. The present study has sought to examine the relationship between fluctuations in air temperature, precipitation, and relative humidity, with a view to providing evidence of potential associations with the transmission dynamics of infectious diseases and the burden of nutritional deficiencies. This is an area that has not been previously explored in Mozambique, and the results of this study highlight the vulnerability of populations in rural areas where environmental instability and limited resources may increase health risks.

Infectious diseases continue to represent the most significant cause of morbidity and mortality, a phenomenon that is particularly evident in low and middle-income countries [15,21], a category that encompasses the study’s setting. The investigation of disease dynamics in these contexts is constrained by a paucity of evidence concerning the role of climate factors in driving these dynamics. Tropical regions are distinguished by their warm climates, which are typified by mild nights and hot days, persistent high humidity, and highly variable precipitation. These conditions are of relevance in the context of understanding patterns of disease transmission.

The findings of the present study demonstrate that among children under five, temperature showed a non-significant association with diarrheal disease risk, responding immediately to extreme heat conditions (RR = 1.20; 95% CI: 0.95–1.45). The influence of risk is further compounded by the quantity of rainfall and the timing of exposure. The most critical threshold is represented by the cumulative effects across successive weeks at higher precipitation levels. This pattern suggests that extremely heavy rainfall may dilute or disperse pathogens and/or alter exposure pathways, lowering the case counts of diarrheal disease. Analogous associations have been observed in other African [37], Asian [12], and Latin American [38] settings. The findings of this study indicate that short-term variations in mean temperature could have been linked to the increase in case counts of childhood diarrheal diseases in Mozambique. However, seasonal patterns may be indicative of broader environmental and behavioral determinants. Consequently, preventive public health measures such as water treatment, sanitation, and hygiene interventions should be prioritized both during periods of extreme weather and in the weeks immediately following, particularly around the threshold when risk peaks.

For malaria, our findings indicate that elevated maximum temperatures may enhance conditions conducive to malaria transmission (RRa = 1.08; 95% CI: 1.01–1.15), potentially through temperature-dependent effects on mosquito vectors and parasite development [4]. This paradox presented in our study, in which malaria-reported weekly case counts increased above 35–40 °C, contradicting the range known to reduce mosquito survival and movement [39]. However, it must be acknowledged that there are inherent limitations to the data available for consideration, as well as the presence of ecological confounding factors. One potential explanation for the observed finding may be attributed to lagged transmission dynamics, the existence of microclimatic refuges that can sustain vector populations, and accelerated parasite development, as well as other behavioral factors that result in an increase in human exposure [3]. Furthermore, wetter weeks are positively and significantly associated with elevated malaria risk (RRa = 1.15; 95% CI: 1.05–1.25) among young children, a finding that can be attributable to the expansion of mosquito breeding habitats [5,40]. This suggests that the impact of extreme precipitation on malaria-reported weekly case counts is immediate and sustained, rather than delayed or sharply fluctuating. Conversely, at elevated precipitation levels, malaria has been observed to decrease, potentially attributable to flooding or water flushing, which disrupts mosquito habitats without eliminating the long-term risk. This finding is consistent with observations in other settings in South-East Asia [5]. These results suggest that malaria transmission is prevalent under conditions of moderate rainfall, while excessive rainfall has the potential to reduce the risk of transmission. Moreover, the present findings serve to reinforce the hypothesis that temperature plays a pivotal role in the dynamics of malaria observed among children under five. In addition, it is posited that humidity and precipitation function as secondary modulators, in accordance with ecological patterns that have been observed in tropical regions.

Despite acknowledging the limitations of raw case counts derived from national health information systems, including potential underreporting, ecological bias, and exposure misclassification, it is imperative to exercise caution when interpreting the results. Nevertheless, the overall pattern observed for both diarrheal disease and malaria underscores the necessity of taking this into consideration when attempting to comprehend disease dynamics [39,41]. Furthermore, it is demonstrated that divergent climatic metrics and disease outcomes manifest distinct temporal risk profiles. The recognition of these patterns has the potential to inform targeted public health interventions such as surveillance and vector control and strengthen early warning systems for climate-sensitive diseases in the studied population, particularly during periods when transmission risk is greatest.

The outcomes of malnutrition have been associated with nutritional habits, which are shaped by the availability and accessibility of agricultural and food products [9,20,42]. In the African context, where this situation is particularly prevalent, additional factors compromise food security [43]. These include social determinants such as conflict, poverty, economic instability, and artisanal mining, as well as environmental determinants such as climate variability and climate change [20,42,44,45]. Furthermore, infectious diseases have been demonstrated to have a substantial impact on adverse nutritional outcomes [10]. Our study demonstrates that moderate acute malnutrition is more prevalent than severe acute malnutrition, with the highest prevalence of 128 cases per one thousand population reported in Manica district. This is consistent with findings from other African settings [2,21,45,46,47]. Furthermore, comparing survey-based data from 2019 to 2023, the prevalence of stunting (highest mean: 39.1 ± 1.8) and underweight (highest mean: 13.9 ± 0.3) is higher than that of wasting (highest mean: 2.3 ± 1.0), a fact that aligns with national patterns reported by INE et al, 2023 [28] and with other studies conducted across the country [48,49,50]. In addition, increased annual mean precipitation (Pmean = 119.3 mm) and lower annual mean temperatures (Tmean = 21.3 °C) reported in our study in 2022, when compared to the years 2019 and 2023, suggest cumulative nutritional deficits over longer periods, which are less responsive to short-term climatic changes. Acknowledging the ecological design of our study, potential confounding factors such as food availability, food access, nutrition habits, and other acute stressors should be explored to avoid misinterpretation of such findings. Comparisons between moderate and severe acute malnutrition across eligible districts and years revealed a negative association between rainfall and moderate acute malnutrition (Spearman’s ρ = −0.652, *p* = 0.0062), while temperature exhibited a similar relationship with severe acute malnutrition (Spearman’s ρ = −0.539, *p* = 0.0312). These findings suggest that years with heavy rainfall are associated with reductions in moderate acute malnutrition, whereas years with extreme temperatures correspond to fewer reported cases of severe acute malnutrition. These findings indicate that climatic variability, specifically reduced precipitation and elevated temperatures, may have a significant negative trend effect on nutritional vulnerability in early childhood. The consistent downward trends observed in both climatic indicators point to the potential influence of environmental stressors on child health outcomes. This pattern indicates that climatic factors influence agricultural production and its variability over time, thereby affecting food access and availability among exposed populations [12]. This, in turn, supports the integration of climate parameters into malnutrition surveillance and resilience planning, emphasizing the necessity of integrating climate indicators into early warning systems.

Notwithstanding the limitations pertaining to data quality, completeness, and the relatively short time series, which compromise the robustness of the analysis, the present study was able to explore potential climatic factors influencing disease dynamics and health outcomes. Moreover, our findings have the potential to inform the health sector about the design and implementation of strategies and interventions informed by environmental and climatic information. Consequently, this will strengthen public health responses in settings exposed to multiple environmental hazards [51,52,53]. The decision to utilize non-parametric correlation analyses with lower statistical power is indicative of the limitations imposed by short and fragmented malnutrition series in our study. Thus, it is imperative that these results be interpreted with caution, as recommended by studies conducted in Ethiopia [54] and Bangladesh [55]. Specifically, our acute malnutrition analysis was limited to 16 annual observations (df = 14) across two districts, and these correlation coefficients represent exploratory associations rather than causal or adjusted epidemiological estimates. A rigorous distinction is maintained between raw case counts, incidence rates per 1000 population at risk, and survey-based prevalence in percentage. For this reason, findings should be cautiously interpreted when drawing definitive causal inferences from these limited time series without the necessity of further longitudinal validation. Consequently, it is imperative that historical surveillance is strengthened, and longitudinal data collection expanded to facilitate future analyses that can more fully characterize the dynamic interplay between climate variability and nutritional outcomes.

In the Revuè sub-basin, the proliferation of artisanal and industrial gold mining has resulted in a substantial encroachment on agricultural potential, as mining operations vie with agricultural activities for land and water resources. Concurrently, these activities result in water and soil waste accumulation, thereby reducing the availability of nutrients and compromising crop productivity [24,56]. Moreover, the introduction of mercury, arsenic, and other contaminants into local rivers has been demonstrated to contribute to chemical and microbiological pollution [57], which has ramifications for food safety and human health. These environmental changes also alter hydrological conditions, creating stagnant water bodies that facilitate vector proliferation, particularly mosquitoes. This could exacerbate malaria transmission risks in mining-affected communities [58]. From an epidemiological modeling perspective, a significant limitation of the GAM/DLNM framework is the omission of dynamic weekly anthropogenic covariates as well as the reliance on raw case counts without a time-varying population offset. Such missing covariates include, but are not limited to, the intensity of artisanal mining, water contamination levels, healthcare access, and seasonal migration flows. Because our models evaluated relative changes in case counts rather than true incidence rates, sudden demographic shifts, such as rapid population influxes due to mining activities or displacement, could theoretically inflate case numbers independently of climate variations. Despite the implementation of control measures to account for slowly evolving socioeconomic and demographic trends through the utilization of smooth functions of calendar time (6–8 df per year) and baseline district population fixed effects, granular weekly time-series data for mining activities and socio-behavioral factors do not currently exist in routine health surveillance systems. The failure to incorporate these non-climatic drivers directly into our regression models underscores the critical need to transition toward integrated One Health surveillance systems that capture both meteorological variables and anthropogenic environmental hazards simultaneously. A paradoxical situation is revealed when an area with abundant natural resources is confronted with concurrent challenges to its agricultural productivity and public health. This finding emerges from a comprehensive consideration of the available data in conjunction with the results of the present study, underscoring the necessity for the formulation of integrated management strategies that can optimize economic gains while ensuring the conservation of ecosystem integrity and the assurance of food security and public health.

The present ecological study found that climate variability was associated with health outcomes [59] in environmentally vulnerable regions, including the risk of diarrheal diseases, malaria, and malnutrition, though its design precludes causal inference. As demonstrated in the extant literature presented in the study, elevated temperatures have been shown to pose a potential risk for malaria. Furthermore, moderate temperatures have been shown to be a contributing factor to the onset of diarrheal diseases. Additionally, interannual precipitation variability has been hypothesized to be associated with elevated levels of moderate acute malnutrition. Conversely, interannual temperature variability was associated with a decline in severe acute malnutrition. It is vital to acknowledge that these vulnerabilities are potentially exacerbated by other environmental hazards, which expose populations to health-adverse risks that need to be explored.

## 5. Conclusions

In this ecological study, we found that climate variability was linked to health outcomes in environmentally vulnerable regions. However, because of the ecological design, these findings point to a pattern rather than a cause-and-effect relationship. While elevated temperatures were consistently associated with increased malaria-reported weekly case counts, moderate temperature ranges contributed to the onset of diarrheal diseases. Interannual precipitation variability and fluctuations in temperature were linked to moderate and severe acute malnutrition, respectively. These findings underscore the complex and non-linear pathways through which climatic factors shape disease burdens and nutritional outcomes. Importantly, such vulnerabilities are likely to be compounded by other environmental hazards, further exposing populations to adverse health risks. Addressing these challenges requires integrated strategies that strengthen resilience within health systems and prioritize climate-sensitive interventions.

## Figures and Tables

**Table 1:** Cumulative relative risk of diarrheal diseases and malaria in four districts, across weekly meteorological indicators (Tmin, Tmax, Tmean, TP and RH) between 2016 and 2024.

Climatic Variables	Tmin (°C)	Tmax (°C)	Tmean (°C)	TP (mm)	RH (%)
**Range (Min–Max)**	7.5–20.0	28.0–36.0	17.5–27.5	0–400	55–85
**Mean ± SD**	14.1 ± 3.8	31.0 ± 3.9	22.6 ± 3.6	91.9 ± 164.7	72.0 ± 9.6
**Risk of diseases**					
**Diarrheal diseases (*n* = 2293)**					
RR (CI 95%)	1.15 (0.90–1.40)	1.20 (0.95–1.45)	1.25 (1.05–1.45) *	1.05 (0.90–1.20)	1.20 (1.00–1.40)
RR lag-adjusted * (CI 95%)	0.97 (0.94–1.00)	0.98 (0.95–1.02)	0.99 (0.96–1.02)	0.98 (0.95–1.02)	1.01 (0.98–1.04)
**Malaria (*n* = 2319)**					
RR (CI 95%)	1.20 (1.05–1.40) *	1.15 (0.90–1.45)	0.95 (0.80–1.10)	1.25 (1.10–1.40) *	0.90 (0.75–1.05)
RR lag-adjusted * (CI 95%)	1.05 (0.98–1.12)	1.08 (1.01–1.15) *	1.02 (1.00–1.05)	1.15 (1.05–1.25) *	1.07 (1.02–1.12) *

* Cumulative relative risk (RR) over the 0–12-week lag period, evaluated at the 90th percentile of weekly meteorological indicators relative to the median reference value. Abbreviations: RH—relative humidity; RR—cumulative risk; SD—standard deviation. Tmin—minimum temperature; Tmax—maximum temperature; Tmean—mean temperature; and TP—total precipitation. * Statistically significant (*p*-value < 0.05).

## Data Availability

The original contributions presented in this study are included in the article. Further inquiries can be directed to the corresponding author(s).

## Appendix A

**Table A1:** Data definition and its respective sources used in the study.

Data	Definition	Unit	Disaggregation	Period	Sources
Acute malnutrition	Defined in children under 5 years as having a weight-for-height or weight-for-length z-score more than 2 SD below the median of the WHO child growth standards, or nutritional edema, or MUAC less than 125 mm. It is subclassified into severe acute malnutrition (SAM) and moderate acute malnutrition (MAM).	Number of cases	annual	2017–2023	DHIS-2
Diarrheal diseases	Defined as the passage of three or more loose or liquid stools per day, caused by infections of the intestinal tract from bacteria, viruses, or parasites.	Number of cases	weekly	2016–2024	DHIS-2/WEB
Malaria	Defined as an infection by Plasmodium parasites transmitted to humans through the bites of infected female Anopheles mosquitoes. Early symptoms are fever, chills, and headache. Infection requires confirmation by rapid testing and/or laboratory identification of the parasites.	Number of cases	weekly	2016–2024	DHIS-2/WEB
Malnutrition indicators	Indicators among children under 5 years old defined as: HAZ—height-for-the-age z-score; WAZ—weight-for-the-age z-score; WHZ—weight-for-the-height z-score.	Percentage	survey	2019, 2022, 2023	FBS, 2019 and 2022 DHS, 2022–2023
Air temperature	Defined as the ambient temperature of the air measured two meters above the surface, expressed in Kelvin and converted to degrees Celsius.	°C	weekly	2016–2024	ECMWF, PSY
Relative humidity	Defined as the ratio of actual water vapor pressure to saturation vapor pressure, expressed as a percentage.	%	weekly	2016–2024	ECMWF, PSY
Total precipitation	Defined as the accumulated sum of convective and large-scale precipitation expressed in units of mm of rainfall.	mm	weekly	2016–2024	ECMWF, PSY

Abbreviations: DHIS-2—routine district-health information system; DHS—Demographic and Health Survey; ECMWF—European Center for Medium-Range Weather Forecasts; FBS—Family Budget Survey; PSY—Provincial Statistics Yearbook; WEB—Weekly Epidemiological Bulletin; mm—millimeters; °C—degrees Celsius; and %—percentage.
